# Associated Comorbidities and Clinical Features of Prolonged Grief Disorder Diagnosis Among Bereaved Individuals: A Retrospective Electronic Health Record (EHR)-Based Cohort Study Using TriNetX

**DOI:** 10.7759/cureus.105320

**Published:** 2026-03-16

**Authors:** Jessica Kuo, Nathan Lebens, Eduardo D Espiridion

**Affiliations:** 1 Psychiatry, Drexel University College of Medicine, Philadelphia, USA

**Keywords:** general & adult psychiatry, grief and bereavement, prolonged grief disorder, psychiatric co-morbidity, utilization of healthcare

## Abstract

Background

Prolonged Grief Disorder (PGD) is a recently recognized psychiatric condition characterized by persistent and debilitating grief following the loss of a loved one. While its clinical relevance is increasingly acknowledged, there is a limited understanding of its epidemiological profile and associated healthcare utilization patterns. This study aimed to characterize the demographic, clinical, and healthcare features of individuals diagnosed with PGD using a large multicenter electronic health record dataset.

Methods

We performed a retrospective cohort analysis using the TriNetX research network (TriNetX, LLC, Cambridge, MA) to identify individuals diagnosed with PGD using the ICD-10 code F43.81. A control cohort was constructed from bereaved individuals without a diagnosis of Prolonged Grief Disorder (PGD) and matched to the PGD cohort on age. Baseline demographic variables (age, sex, race, ethnicity), psychiatric diagnoses, substance use disorders, and emergency department (ED) utilization were extracted and compared between cohorts. Statistical comparisons between cohorts were conducted using chi-squared tests for categorical variables and independent samples t-tests for continuous variables.

Results

The PGD cohort included 2,464 participants, of whom 1,836 (74.5%) were female and 1,755 (71.2%) were White, with a mean age of 59.2 years. The control cohort comprised 91,868 participants. Depressive episodes were present in 1,995 (81.0%) PGD individuals and 49,835 (54.3%) controls. Recurrent major depressive disorder was identified in 1,370 (55.6%) PGD individuals and 24,795 (27.0%) controls. Other anxiety disorders were observed in 1,988 (80.7%) PGD individuals and 55,802 (60.8%) controls. Alcohol-related disorders were documented in 598 (24.3%) PGD individuals and 16,906 (18.4%) controls, while nicotine dependence occurred in 964 (39.1%) PGD individuals and 29,636 (32.3%) controls. Emergency department utilization was higher among PGD individuals, occurring in 1,940 (78.7%) individuals and 65,385 (71.2%) controls. Differences across nearly all diagnostic categories were statistically significant.

Conclusions

Individuals with Prolonged Grief Disorder demonstrate a substantially elevated burden of psychiatric comorbidities and increased healthcare utilization. These findings reinforce the importance of recognizing PGD as a clinically meaningful diagnosis with significant implications for mental health services. Future research is needed to evaluate treatment strategies and long-term outcomes for this population.

## Introduction

Prolonged Grief Disorder (PGD) is a psychiatric diagnosis describing a form of bereavement in which grief remains persistent and functionally impairing rather than gradually diminishing over time. Although grief is a universal human experience, a subset of bereaved individuals develop enduring yearning for the deceased, intense emotional pain, disruptions in identity, and sustained impairment that extends beyond culturally expected periods of mourning. Diagnostic criteria for PGD were developed through longitudinal and psychometric work identifying a reliable symptom profile [1], contributing to its inclusion in the Diagnostic and Statistical Manual of Mental Disorders, Fifth Edition, Text Revision (DSM-5-TR) [2] and the International Classification of Diseases, 11th Revision (ICD-11) [3]. Formal recognition of PGD has increased attention to the condition as a distinct clinical syndrome requiring targeted assessment and intervention.

Epidemiological research suggests that PGD affects a substantial minority of bereaved adults. An early meta-analysis of adult bereavement samples estimated a pooled prevalence of approximately 10% [4]. More recent cross-national meta-analytic studies report pooled estimates in the range of 10-15%, with considerable heterogeneity across studies [5]. In this context, heterogeneity refers to variability in prevalence estimates across studies, which may reflect differences in sampling strategies, age composition, diagnostic criteria, and sociocultural context. Variation in prevalence has been associated with differences in sampling strategies, age composition, diagnostic criteria, and sociocultural context, underscoring how methodological and contextual factors influence observed rates. Nonetheless, available data consistently indicate that PGD represents a clinically significant and potentially high-burden condition among bereaved adults.

PGD frequently co-occurs with other psychiatric disorders, most notably major depressive disorder (MDD) and posttraumatic stress disorder (PTSD). Population-based studies demonstrate substantial comorbidity among bereaved adults meeting criteria for PGD [6]. However, factor-analytic investigations suggest that PGD symptoms cluster separately from depressive and trauma-related symptom dimensions [7], supporting PGD as a distinct diagnostic entity rather than a variant of depression or trauma-related disorders.

Despite increasing recognition of PGD, important gaps remain in the literature. Much of the existing research has relied on community-based samples and self-report screening instruments rather than clinically coded diagnoses, which may limit understanding of how PGD presents in real-world healthcare settings. In addition, many prior studies have been conducted in relatively small or geographically limited samples, restricting the ability to characterize large-scale patterns of psychiatric comorbidity and healthcare utilization. Although PGD is now formally recognized and captured in electronic health records using ICD-10-CM code F43.81, large multicenter analyses examining individuals diagnosed with PGD in routine clinical practice remain limited.

To address these gaps, the present study uses a large multicenter electronic health record (EHR) network to examine the clinical characteristics and healthcare utilization patterns of adults diagnosed with Prolonged Grief Disorder in routine clinical practice. Using the TriNetX research network, we conducted a retrospective cohort analysis comparing adults with a documented diagnosis of PGD to age-matched bereaved controls without PGD. The primary objective of this study was to characterize differences between these groups across three clinically relevant domains: (1) psychiatric comorbidities, including depressive, anxiety, bipolar, psychotic, and suicide-related diagnoses; (2) substance use disorders; and (3) healthcare utilization, specifically emergency department visits. We hypothesized that adults diagnosed with PGD would demonstrate a greater burden of psychiatric comorbidity, higher prevalence of substance use disorders, and increased emergency department utilization compared with bereaved controls without PGD.

## Materials and methods

This retrospective record review was conducted using data from the TriNetX Research Network (TriNetX, LLC, Cambridge, MA), a federated research platform aggregating de-identified electronic health records (EHRs) from participating healthcare organizations across the United States. All analyses were performed within the TriNetX secure environment in compliance with the Health Insurance Portability and Accountability Act (HIPAA) and institutional privacy regulations. Because all data were fully de-identified, this study was exempt from institutional review board approval.

Study population

Records of adults aged 18 years and older with a documented diagnosis of bereavement were eligible for inclusion. Bereavement was defined using ICD-10-CM code Z63.4 (“Disappearance and death of family member”). The study period extended from January 1, 2015, to March 25, 2025. The prolonged grief disorder cohort consisted of individuals with a subsequent diagnosis of PGD (ICD-10-CM F43.81) occurring after the initial bereavement diagnosis. The control cohort consisted of individuals with a bereavement diagnosis (ICD-10-CM code Z63.4) and no documented PGD diagnosis during the available follow-up period captured in the database. Control individuals were required to have at least one healthcare encounter following bereavement. For individuals in the PGD cohort, the PGD diagnosis (ICD-10-CM code F43.81) was required to occur after the bereavement index date, thereby enforcing temporal ordering between bereavement and PGD when present.

Records were excluded if the PGD diagnosis preceded the bereavement diagnosis or if either code lacked a valid encounter date. All individuals were required to have at least one healthcare encounter following bereavement. The index date was defined as the earliest encounter associated with the Z63.4 diagnosis. For individuals in the PGD cohort, the F43.81 diagnosis was required to occur after the index date. Control individuals were required to have no documented PGD diagnosis at any time during the available observation period following the bereavement index encounter.

Study variables

Variables include demographic characteristics such as age at index date, sex, race, and ethnicity. Psychiatric and behavioral health diagnoses were identified using ICD-10-CM codes, including: Depressive episode (F32), Recurrent major depressive disorder (F33), Other anxiety disorders (F41), Phobic anxiety disorders (F40), Obsessive-compulsive disorder (F42), Bipolar disorder (F31), Schizophrenia (F20), Schizoaffective disorder (F25), and Suicide attempt (T14.91). Substance-related disorders included alcohol (F10), nicotine (F17), cannabis (F12), opioid (F11), cocaine (F14), and sedative/anxiolytic use (F13) disorders. Diagnoses were considered present if documented at any time after the index encounter. Emergency department utilization was evaluated as a binary outcome, defined as the presence of at least one ED visit following bereavement.

Statistical analysis

Descriptive statistics were used to summarize demographic and clinical characteristics. Categorical variables were reported as frequencies and percentages, while continuous variables were expressed as means. Between-group comparisons were performed using chi-square tests for categorical variables and independent samples t-tests for continuous variables. Odds ratios (ORs) with 95% confidence intervals (CIs) were calculated for binary outcomes to quantify effect size. Due to platform limitations within the TriNetX analytics environment, multivariable regression and time-to-event analyses were not performed. Accordingly, all comparisons are unadjusted and should be interpreted as descriptive associations rather than independent effect estimates. All statistical analyses were conducted using the TriNetX web-based analytics platform. A two-sided p-value < 0.05 was considered statistically significant.

## Results

Study population and demographic characteristics

From January 1, 2015, to March 25, 2025, a total of 2,464 individuals diagnosed with Prolonged Grief Disorder (PGD) were identified and compared with an age-matched control cohort of 91,868 individuals without PGD. The mean age of 59.2 years was identical between groups. The PGD cohort included 1,836 (74.5%) females and 628 (25.5%) males, while the control cohort included 61,838 (67.3%) females and 29,996 (32.7%) males. Female sex was more prevalent in the PGD group (p < 0.0001), and male sex was more common among controls (p < 0.0001). Among PGD individuals, 1,755 (71.2%) were White, 470 (19.1%) were Black or African American, 44 (1.8%) were Asian, 23 (0.9%) were American Indian or Alaska Native, 17 (0.7%) were Native Hawaiian or Other Pacific Islander, 80 (3.2%) were of other race, and 75 (3.0%) had unknown race. White race was modestly more prevalent among PGD individuals (p = 0.0021), while Asian race was slightly less common (p = 0.0233). No significant differences were observed for other racial categories. Ethnicity distribution in the PGD cohort included 1,801 (73.1%) individuals identified as not Hispanic or Latino, 233 (9.5%) as Hispanic or Latino, and 430 (17.4%) as unknown, with no significant differences compared with controls (all p > 0.05).

Psychiatric comorbidities

Psychiatric disorders were highly prevalent among individuals with PGD (Table 1). Depressive episodes (F32) were documented in 1,995 (81.0%) individuals with PGD and 49,835 (54.3%) controls (OR 3.59, 95% CI 3.23-3.99; p < 0.0001). Recurrent major depressive disorder (F33) was present in 1,370 (55.6%) individuals with PGD and 24,795 (27.0%) controls (OR 3.39, 95% CI 3.12-3.69; p < 0.0001). Other anxiety disorders (F41) were observed in 1,988 (80.7%) individuals with PGD and 55,802 (60.8%) controls (OR 2.70, 95% CI 2.42-3.02; p < 0.0001). Phobic anxiety disorders (F40) were identified in 226 (9.2%) individuals with PGD and 4,226 (4.6%) controls (OR 2.09, 95% CI 1.81-2.42; p < 0.0001). Obsessive-compulsive disorder (F42) was documented in 106 (4.3%) individuals with PGD and 2,382 (2.6%) controls (OR 1.69, 95% CI 1.38-2.07; p <0.0001). Bipolar disorder (F31) was more prevalent among individuals with PGD, occurring in 388 (15.7%) individuals compared with 10,059 (10.9%) controls (OR 1.52, 95% CI 1.36-1.69; p < 0.0001). Manic episodes (F30) were infrequent and observed in 37 (1.5%) individuals with PGD and 1,019 (1.1%) controls (OR 1.36, 95% CI 0.96-1.93; p = 0.0677). Schizophrenia (F20) was documented in 88 (3.6%) individuals with PGD and 2,814 (3.1%) controls (OR 0.91, 95% CI 0.73-1.14), while schizoaffective disorder (F25) occurred in 84 (3.4%) individuals with PGD and 3,071 (3.3%) controls (OR 1.02, 95% CI 0.81-1.28). Suicide attempts (T14.91) were recorded in 156 (6.3%) individuals with PGD and 2,531 (2.8%) controls (OR 2.40, 95% CI 2.02-2.85; p < 0.0001).

**Table 1:** Comparison of baseline demographics and clinical characteristics between the PGD and control cohorts. Continuous variables (age) were compared using independent samples t-tests, and corresponding t-values are reported alongside p-values. Categorical variables were compared using chi-square tests of independence, and corresponding chi-square values are reported alongside p-values. A two-tailed p-value < 0.05 was considered statistically significant. Superscript symbols denote the statistical tests used for each comparison. * indicates p-values derived from independent samples t-tests, while # denotes the corresponding t-statistic (t value) for that comparison. Effect sizes were calculated as Odds Ratios (OR) with 95% Confidence Intervals (CI).

Variable	PGD Count	PGD %	Control Count	Control %	Chi-Square Value	Chi-Square P-Value	OR (95% CI)
Age (Mean ± SD)	59.2 ± 18.4	100%	59.2 ± 16.2	100%	0.00#	0.9555*	N/A
Female	1836	74.5%	61838	67.3%	56.35	< 0.0001	1.42 (1.30–1.54)
Male	628	25.5%	29996	32.7%	56.35	< 0.0001	0.70 (0.65–0.77)
Not Hispanic or Latino	1801	73.1%	66861	72.8%	0.10	0.7304	1.02 (0.94–1.10)
Unknown Ethnicity	430	17.4%	16896	18.4%	1.44	0.2342	0.94 (0.85–1.05)
Hispanic or Latino	233	9.5%	8111	8.8%	1.16	0.2792	1.08 (0.94–1.24)
White	1755	71.2%	62749	68.3%	9.32	0.0021	1.15 (1.06–1.25)
Black or African American	470	19.1%	18247	19.9%	0.95	0.3334	0.95 (0.86–1.05)
Other Race	80	3.2%	3642	4.0%	3.27	0.0710	0.81 (0.64–1.02)
Asian	44	1.8%	2303	2.5%	5.16	0.0233	0.70 (0.51–0.94)
Unknown Race	75	3.0%	3472	3.8%	3.60	0.0582	0.78 (0.61–1.00)
American Indian or Alaska Native	23	0.9%	946	1.0%	0.22	0.6399	0.90 (0.59–1.37)
Native Hawaiian or Other Pacific Islander	17	0.7%	509	0.6%	0.80	0.3714	1.24 (0.75–2.03)
F32 - Depressive episode	1995	81.0%	49835	54.3%	691.05	< 0.0001	3.60 (3.28–3.95)
F33 - Major depressive disorder, recurrent	1370	55.6%	24795	27.0%	979.10	< 0.0001	3.38 (3.12–3.66)
F41 - Other anxiety disorders	1988	80.7%	55802	60.8%	401.23	< 0.0001	2.70 (2.47–2.96)
F40 - Phobic anxiety disorders	226	9.2%	4226	4.6%	111.42	< 0.0001	2.10 (1.82–2.42)
F42 - Obsessive-compulsive disorder	106	4.3%	2382	2.6%	27.26	<0.0001	1.69 (1.37–2.08)
F17 - Nicotine dependence	964	39.1%	29636	32.3%	51.40	< 0.0001	1.34 (1.23–1.46)
F10 - Alcohol-related disorders	598	24.3%	16906	18.4%	54.51	< 0.0001	1.42 (1.29–1.56)
F12 - Cannabis-related disorders	433	17.6%	11455	12.5%	56.64	< 0.0001	1.49 (1.34–1.66)
F11 - Opioid-related disorders	308	12.5%	8184	8.9%	37.70	< 0.0001	1.46 (1.29–1.65)
F14 - Cocaine-related disorders	201	8.2%	5190	5.6%	27.96	< 0.0001	1.49 (1.28–1.73)
Sedative/anxiolytic disorders	144	5.8%	2814	3.1%	61.03	< 0.0001	1.96 (1.64–2.35)
F20 - Schizophrenia	88	3.6%	3585	3.9%	0.71	0.4021	0.91 (0.73–1.13)
F25 - Schizoaffective disorders	84	3.4%	3071	3.3%	0.03	0.8568	1.02 (0.81–1.29)
F31 - Bipolar disorder	388	15.7%	10059	10.9%	55.96	< 0.0001	1.53 (1.37–1.71)
F30 - Manic episode	37	1.5%	1019	1.1%	3.33	0.0677	1.36 (0.96–1.91)
T14.91 - Suicide attempt	156	6.3%	2531	2.8%	110.79	< 0.0001	2.39 (2.01–2.84)
Emergency Department Visits	1940	78.7%	65385	71.2%	66.71	< 0.0001	1.49 (1.35–1.64)

Substance use disorders

Substance use disorders were more prevalent among individuals with PGD (Table 1). Nicotine dependence (F17) was present in 964 (39.1%) individuals with PGD and 29,636 (32.3%) controls (OR 1.35, 95% CI 1.25-1.47; p < 0.0001). Alcohol-related disorders (F10) were observed in 598 (24.3%) individuals with PGD and 16,906 (18.4%) controls (OR 1.42, 95% CI 1.29-1.55; p < 0.0001). Cannabis-related disorders were documented in 433 (17.6%) individuals with PGD and 11,455 (12.5%) controls (OR 1.50, 95% CI 1.35-1.67; p < 0.0001), opioid-related disorders in 308 (12.5%) individuals with PGD and 8,184 (8.9%) controls (OR 1.45, 95% CI 1.27-1.65; p < 0.0001), cocaine-related disorders in 201 (8.2%) individuals with PGD and 5,190 (5.6%) controls (OR 1.49, 95% CI 1.28-1.74; p < 0.0001), and sedative/anxiolytic-related disorders in 144 (5.8%) individuals with PGD and 2,814 (3.1%) controls (OR 1.96, 95% CI 1.64-2.33; p < 0.0001).

Healthcare utilization

Emergency department utilization was higher among individuals with PGD (Table 1). At least one ED visit following bereavement was documented in 1,940 (78.7%) individuals with PGD and 65,385 (71.2%) controls (OR 1.50, 95% CI 1.36-1.66; p < 0.0001).

## Discussion

Individuals with a documented ICD-10-CM diagnosis of Prolonged Grief Disorder (F43.81) demonstrated higher documented rates of psychiatric comorbidity, substance use disorders, and emergency department utilization compared with age-matched bereaved adults without PGD. The largest effect sizes were observed for depressive and anxiety disorders, with odds ratios ranging from 2.10 to 3.59, whereas differences in psychotic disorders were minimal. Suicide attempts were also meaningfully elevated, with more than twofold higher odds among individuals with PGD, while substance-related disorders and emergency department utilization showed more modest but consistent increases. These findings suggest that, although many comparisons were statistically significant, the magnitude of association varied across outcomes and was greatest for mood- and anxiety-related conditions. Overall, this pattern aligns with conceptual models that position PGD primarily within the stress- and mood-related spectrum of psychiatric disorders.

Psychiatric comorbidity

The high prevalence of depressive and anxiety disorders among individuals with PGD observed in this cohort is consistent with prior research demonstrating substantial psychiatric overlap in bereaved populations. In the present study, individuals with PGD had substantially higher odds of depressive episodes (OR 3.59, 95% CI 3.23-3.99), recurrent major depressive disorder (OR 3.39, 95% CI 3.12-3.69), and anxiety disorders (OR 2.70, 95% CI 2.42-3.02) compared with bereaved controls. Population-based studies have reported frequent co-occurrence between PGD, major depressive disorder, and posttraumatic stress disorder [6]. At the same time, psychometric analyses suggest that PGD symptoms remain empirically distinct from depressive and trauma-related symptom clusters [7]. The current findings extend this literature by demonstrating similar patterns within a large, multicenter clinical population identified through routine diagnostic coding rather than research-based self-report instruments.

From a clinical perspective, these results highlight the importance of comprehensive psychiatric assessment when evaluating bereaved individuals presenting with persistent grief symptoms. Clinicians should consider screening not only for PGD but also for co-occurring depressive and anxiety disorders, which appear to represent the most prominent components of the psychiatric burden associated with the condition. Integrated treatment approaches addressing both grief-specific symptoms and broader mood or anxiety pathology may therefore be particularly relevant in this population.

Suicide attempts

Suicide attempts were more frequently documented among individuals with PGD compared with bereaved controls. Suicide attempts were recorded in 156 (6.3%) individuals with PGD and 2,531 (2.8%) controls, corresponding to more than twice the odds of a documented suicide attempt among individuals with PGD (OR 2.40, 95% CI 2.02-2.85). Prior research has consistently linked prolonged grief symptoms to elevated suicidal ideation and behaviors. A recent meta-analysis and systematic review reported substantial rates of suicidal ideation, suicide attempts, and suicide deaths among individuals meeting criteria for PGD [8]. In addition, other studies have found that PGD symptom severity is independently associated with recent suicidal ideation among bereaved individuals, even when accounting for depressive symptoms [9]. The present findings indicate that the difference in ED utilization, while modest in magnitude, corresponded to approximately 1.5-fold higher odds of emergency department utilization among individuals with PGD (OR 1.50, 95% CI 1.36-1.66). Given the large sample size, this distinction between statistical significance and effect size is important; the observed association likely reflects a real but moderate increase in acute-care use rather than a dramatic difference in utilization patterns.

These findings have important implications for clinical care. Bereaved individuals presenting with persistent grief symptoms may warrant routine suicide risk assessment, particularly when accompanied by depressive symptoms or functional impairment. Early identification of individuals at elevated risk could facilitate timely referral to mental health services and targeted interventions designed to reduce suicide risk among vulnerable bereaved individuals.

Substance use disorder

Substance use disorders were also more prevalent among individuals with PGD across multiple categories, including alcohol-, nicotine-, cannabis-, opioid-, cocaine-, and sedative/anxiolytic-related diagnoses. Across substance categories, odds ratios ranged from 1.35 to 1.96, indicating modest but consistent elevations in documented substance-related diagnoses among individuals with PGD. Prior work examining complicated grief, much of which predates the formal recognition of PGD in DSM-5-TR and ICD-11, has identified a positive association between grief-related psychopathology and substance misuse. A systematic review by Parisi et al. found evidence of bidirectional associations, with substance misuse increasing risk for subsequent prolonged or complicated grief symptoms and elevated grief severity predicting increases in smoking and alcohol dependence [10]. These findings underscore the need for screening for substance use disorders in individuals presenting with PGD, as well as the potential value of integrated behavioral health approaches that address grief symptoms alongside substance-related concerns.

Emergency department visits

Emergency department utilization was more common among individuals diagnosed with PGD. While prior research demonstrates that bereavement may transiently increase healthcare use in the months following loss [11] and that persistently elevated grief symptoms are associated with sustained medical visits and excess mortality over longer follow-up periods [12], these studies did not evaluate individuals identified through formal PGD diagnostic coding. Additionally, smaller clinical samples suggest that individuals meeting PGD criteria do not consistently engage structured outpatient mental health services [13]. The present findings indicate that the difference in ED utilization, while statistically significant, corresponds to approximately 1.5-fold higher odds of emergency department utilization among individuals with PGD (OR 1.50, 95% CI 1.36-1.66).

From a health systems perspective, these findings highlight the potential role of emergency departments as points of clinical contact for bereaved individuals experiencing significant psychological distress. Improving screening and referral pathways for grief-related disorders in acute care settings may help connect individuals with appropriate outpatient mental health services. Early recognition of PGD within healthcare systems could facilitate referral to evidence-based grief-focused treatments and potentially reduce reliance on acute care services over time.

Limitations

Several limitations should be considered when interpreting these findings. First, the study relied on ICD-10-CM diagnostic codes recorded in electronic health records, which introduces the possibility of misclassification bias. Diagnostic coding practices may vary across clinicians and institutions, and psychiatric conditions such as prolonged grief disorder may be underrecognized or inconsistently documented in routine clinical care. Because PGD is a relatively recent diagnostic addition, clinician familiarity with the diagnosis and its coding may still be evolving. As a result, some individuals who meet clinical criteria for PGD may not receive a formal diagnosis, while others may be coded inaccurately. Similarly, psychiatric comorbidities, substance use disorders, and suicide attempts may be underdocumented if they are not formally recorded in structured diagnostic fields.

Second, the analysis was based on unadjusted comparisons between cohorts and did not include multivariable regression modeling to control for potential confounding factors. Variables such as sex, race, prior psychiatric history, healthcare access, and other clinical or social characteristics could influence both the likelihood of receiving a PGD diagnosis and the presence of psychiatric comorbidities or healthcare utilization. The inability to perform multivariable adjustment limits the extent to which the observed associations can be interpreted as independent of these potential confounders.

Third, psychiatric and substance-related diagnoses were considered present if documented at any time following bereavement and were not restricted to incident diagnoses occurring after the PGD diagnosis. Consequently, the temporal relationship between PGD and comorbid psychiatric conditions cannot be established. These diagnoses may represent pre-existing vulnerability, concurrent psychiatric burden, or potential sequelae of prolonged grief.

Fourth, emergency department utilization was captured as a binary indicator without information on visit frequency, timing relative to bereavement, or the specific reasons for presentation. This limits the interpretation of the clinical drivers of increased acute-care utilization.

Finally, the cohort was derived from individuals receiving care within healthcare systems contributing data to the TriNetX network and was defined by the presence of a bereavement diagnosis code (Z63.4). This approach likely captures a subset of bereaved individuals who are already engaged in healthcare services and may not fully represent bereavement experiences in community populations. In addition, because PGD diagnostic coding is still being adopted in clinical practice, some individuals in the control group may have had unrecognized or uncoded PGD, which would likely bias observed differences toward the null.

## Conclusions

A documented PGD diagnosis appears to identify a population with elevated psychiatric comorbidity, higher documented suicide attempt rates, and increased substance-related disorders. In practice, this suggests that identification of PGD should prompt careful suicide risk assessment, evaluation for co-occurring depressive and anxiety disorders, and screening for substance use disorders. The elevated emergency department utilization observed in this cohort further underscores the need for proactive outpatient management and coordinated care. Longitudinal research with more granular utilization data will be necessary to determine whether early identification and integrated intervention strategies improve clinical outcomes in this population. These real-world data reinforce PGD as a clinically meaningful diagnosis and highlight the need for continued investigation into its course, service needs, and long-term outcomes.
